# Advances in natural products and antibody drugs for SLE: new therapeutic ideas

**DOI:** 10.3389/fphar.2023.1235440

**Published:** 2023-07-10

**Authors:** Yibing Han, Lingwei Liu, Bo Zang, Ruiwen Liang, Xinyue Zhao, Bin Liu

**Affiliations:** Department of Rheumatology, The Affiliated Hospital of Qingdao University, Shandong, China

**Keywords:** systemic lupus erythematosus, antibody drug, natural products, treatment, immune system

## Abstract

Systemic Lupus Erythematosus (SLE) is a chronic autoimmune systemic disease with a wide range of clinical symptoms, complex development processes, and uncertain prognosis. The clinical treatment of SLE is mainly based on hormones and immunosuppressants. Research on novel therapy strategies for SLE has flourished in recent years, especially the emergence of new targeted drugs and natural products that can modulate related symptoms. This review discusses the current experience including B-cell targeted drugs (belimumab, tabalumab, blisibimod, atacicept, rituximab, ofatumumab, ocrelizumab, obexelimab, and epratuzumab), T-cell targeted drugs (abatacept, dapirolizumab, and inhibitor of syk and CaMKIV), cytokines targeted drugs (anifrolumab and sifalimumab), and natural products (curcumin, oleuropein, punicalagin, sulforaphane, icariin, apigenin, and resveratrol). The aim of this paper is to combine the existing *in vitro* and *in vivo* models and clinical research results to summarize the efficacy and mechanism of natural drugs and targeted drugs in SLE for the reference and consideration of researchers.

## 1 Introduction

SLE is a complex autoimmune inflammatory disease with a diverse course and prognosis that can involve multiple organs throughout the body. It mostly affects women, and its clinical symptoms range from mild to life-threatening. This clinical heterogeneity is most likely caused by intricate immunological dysregulation, like the loss of immunological tolerance to autoantigens and the development of multiple autoantibodies (Bentham et al., 2015). Immune complexes formed by autoantibodies binding to intracellular autoantigens are deposited in the skin, blood vessels, kidneys, and liver. Over time, these immune complexes will cause tissue damage and the emergence of a variety of diseases or manifestations, such as zygomatic rash, arthralgia, fever, renal failure, and cardiovascular disease (Fava and Petri, 2019; Herrada et al., 2019). Lupus nephritis (LN) is the primary clinical symptom of SLE. It may have a negative impact on the life quality and long-term prognosis of SLE patients (Davidson, 2016). Roughly 50% of SLE patients will eventually acquire renal dysfunction (Almaani et al., 2017).

The disease cannot be cured for now, but its progression can be controlled by early diagnosis and medications. Currently, the drugs used to treat SLE include antimalarial therapy, glucocorticoids (GC), non-steroidal anti-inflammatory drugs (NSAIDs), and immunosuppressants including azathioprine (AZA), cyclophosphamide (CYC), tacrolimus (TAC), and mycophenolate mofetil (MMF). However, conventional treatments are accompanied by significant side effects and ideal treatment options are rare. The anti-inflammatory effects of these medicines are accompanied by adverse effects caused by the toxic effects of the drug, including cataracts, osteoporotic fractures, cardiovascular injury, severe infections, malignancies, teratogenicity, and infertility, potentially leading to additional organ damage and mortality (Curtis et al., 2006; Apostolopoulos and Morand, 2017; Ponticelli and Moroni, 2017; Deng et al., 2019; Felten et al., 2019). Therefore, The medical community is eager for safer treatment profiles and more targeted therapies.

Fortunately, an increasing number of studies have focused on exploring new drugs and therapies and found that targeted drugs and natural products offer significant therapeutic promise in the treatment of SLE.

## 2 Targeted agents

Due to the low specificity and adverse effects of traditional therapeutic agents, there is still some unmet need for more targeted agents with better safety profiles in SLE treatment. Based on recent advances in understanding the complex pathogenesis of SLE, several targeted therapies are presently being evaluated in clinical trials.

### 2.1 Targeted drugs against B lymphocytes

A wealth of evidence points to B cells as key players in the pathogenesis of SLE. B cells initiate self-reactive T cells, such as antigen-presenting cells, to release pro-inflammatory cytokines and chemokines, promote the generation of autoimmune responses in target organs, and are considered as potential targets for SLE therapy (Table 1) (Sanz and Lee, 2010; Tsokos et al., 2016).

**TABLE 1:** B-cell-targeted investigation for treatment of SLE.

Medicine	Mechanism of action	Trial	Trial phase	SLE population	Method of administration	Significant findings	Ref
Belimumab	Monoclonal antibody (IgG1) targeted soluble BAFF	BLISS-52	Ⅲ	SELENA-SLEDAI≥6	IV	Significantly higher SRI rate with belimumab 1 mg/kg and 10 mg/kg than with placebo	Navarra et al. (2011)
				Positive for anti-dsDNA or ANA			
		BLISS-76	Ⅲ	SELENA-SLEDAI≥6	IV	Significantly greater SRI response with belimumab 10 mg/kg at week 52 compared with placebo. There was no difference between groups when followed to 76 weeks	Furie et al. (2011)
				Positive for anti-dsDNA or ANA			
		BLISS-LN	Ⅲ	LN	IV	Significantly more patients in the belimumab group (10 mg/kg) had a primary efficacy renal response and a complete renal response than in the placebo group at week 104	Furie et al. (2020)
		BLISS-LN	Ⅲ	LN	IV	Increases occurred in the proportions of patients achieving primary efficacy renal response and complete renal response from open-label baseline to week 28	Furie et al. (2022)
Tabalumab	Monoclonal antibody (IgG4) targeted soluble and membrane BAFF	ILLUMINATE-1	Ⅲ	SELENA-SLEDAI≥6	SC	No significant difference in SRI-5, time to first flare, or steroid-sparing effect in tabalumab-treated patients. Significant reductions in anti-dsDNA antibodies, increase in C3 and C4, and reductions in total B cells and immunoglobulins were observed with tabalumab	Isenberg et al. (2016)
				ANA positive			
		ILLUMINATE-2	Ⅲ	SELENA-SLEDAI≥6	SC	Primary endpoint was met with 120 mg every 2 weeks. Key secondary endpoints were not met.	Merrill et al. (2016)
				ANA positive			
Blisibimod	Peptibody with 4 BAFF-binding targeted BAFF	PEARL-SC	Ⅱ	SELENA-SLEDAI≥6	SC	SRI-5 response rates were not significantly improved in the pooled blisibimod groups. Significant changes in anti-dsDNA antibodies, complement C3 and C4, and reductions in B cells were observed with blisibimod	Furie et al. (2015)
				Positive for anti-dsDNA or ANA			
		CHABLIS-SC1	Ⅲ	SELENA-SLEDAI≥6	SC	SRI-6 primary endpoint was not met. A significant steroid-sparing effect appeared with blisibimod-treated subjects and achieved corticosteroid taper. Blisibimod was associated with successful steroid reduction, decreased proteinuria and biomarker responses	Merrill et al. (2018a)
				Systemic corticosteroid			
				Positive for anti-dsDNA or ANA			
Atacicept	A fusion protein of TACI and Fc domain of human IgG1 targeted both BAFF and April	ADDRESS Ⅱ	Ⅱb	SLEDAI-2K ≥ 6	SC	The SRI-4 response rate was improved with atacicept 75mg and 150 mg at week 24 as compared with placebo. The *post hoc* analysis showed that only the cohort with high disease activity at baseline did meet the primary endpoint in patients receiving atacicept 150 mg	Merrill et al. (2018b), Morand et al. (2020b), Wallace et al. (2021)
				Positive for anti-dsDNA or ANA			
Rituximab	Monoclonal antibody targeted surface CD20	EXPLORE	Ⅱ/Ⅲ	≥1 BILAG A score or ≥2 BILAG B scores	IV	No differences were noted between placebo and rituximab in the primary and secondary endpoints	Merrill et al. (2010b)
				Positive for ANA			
				Stable use of 1 immunosuppressive drug			
		LUNAR	Ⅲ	Class Ⅲ or Ⅳ LN according to the 2003 ISN/RPS	IV	The primary endpoint was not achieved. Statistically significant improvements in serum complement C3, C4 and anti-dsDNA level and reduction of proteinuria were observed among patients treated with rituximab	Rovin et al. (2012)
				Positive for ANA			
Ofatumumab	Monoclonal antibody targeted surface CD20	Case series	-	Treated with ofatumumab for SLE/LN since 2012	IV	B cell depletion was achieved in 12/14 patients, serological markers of disease activity have improved. Half of the patients with LN achieved renal remission by 6 months. Ofatumumab is a useful alternative in rituximab-allergic patients	Masoud et al. (2018)
Ocrelizumab	Monoclonal antibody targeted surface CD20	BELONG	Ⅲ	Positive for ANA	IV	Overall renal response rates with ocrelizumab in patients with active LN were numerically superior to those with placebo. But ocrelizumab treatment was associated with a higher rate of serious infections	Mysler et al. (2013)
				Active LN			
Obexelimab	Monoclonal antibody targeted surface CD19 and increased affinity for FcγRIIb	-	Ⅱ	SLEDAI decrease ≥4 points or ≥1 grade decrease in ≥1 BILAG A or B score	IV	No significantly difference between groups in an efficacy evaluable population analysis. There was a significant difference in the intent to treat analysis at 169 days, but not at 225 days	Merrill et al. (2018c)
Epratuzumab	Monoclonal antibody targeted surface CD22	EMBLEM	Ⅱb	BILAG 2004 of grade A in ≥1 organ/system or grand B in ≥2 organs/systems	IV	Proportion of responders was higher in all epratuzumab groups than with placebo	Wallace et al. (2014)
				SLEDAI-2K ≥ 6			
		EMBODY 1 & 2	Ⅲ	Positive for anti-dsDNA or ANA	IV	No statistically significant difference in the primary end point between the groups	Clowse et al. (2017)
				SLEDAI-2K ≥ 6			
				BILAG-2004 scores of grade A in ≥1 body system or grand B in ≥2 body system			

Abbreviations: SLE: systemic lupus erythematosus; BAFF: B-cell-activation factor; TACI: transmembrane activator calcium moderator and cyclophilin lingand interactor; April: a proliferation-inducing ligand; BLISS: belimumab in subjects with SLE; IV: intravenous; SELENA-SLEDAI: Safety of Estrogens in Lupus Erythematosus National Assessment–Systemic Lupus Erythematosus Disease Activity Index; dsDNA: double-stranded DNA; ANA: antinuclear antibodies; SRI: SLE, responder index; LN: lupus nephritis; SC: subcutaneous; SLEDAI-2K: SLE, disease activity index 2000; BILAG: british isles lupus assessment; ISN/RPS: International Society of Nephrology/Renal Pathology Society.

#### 2.1.1 Belimumab

**FIGURE 1:**
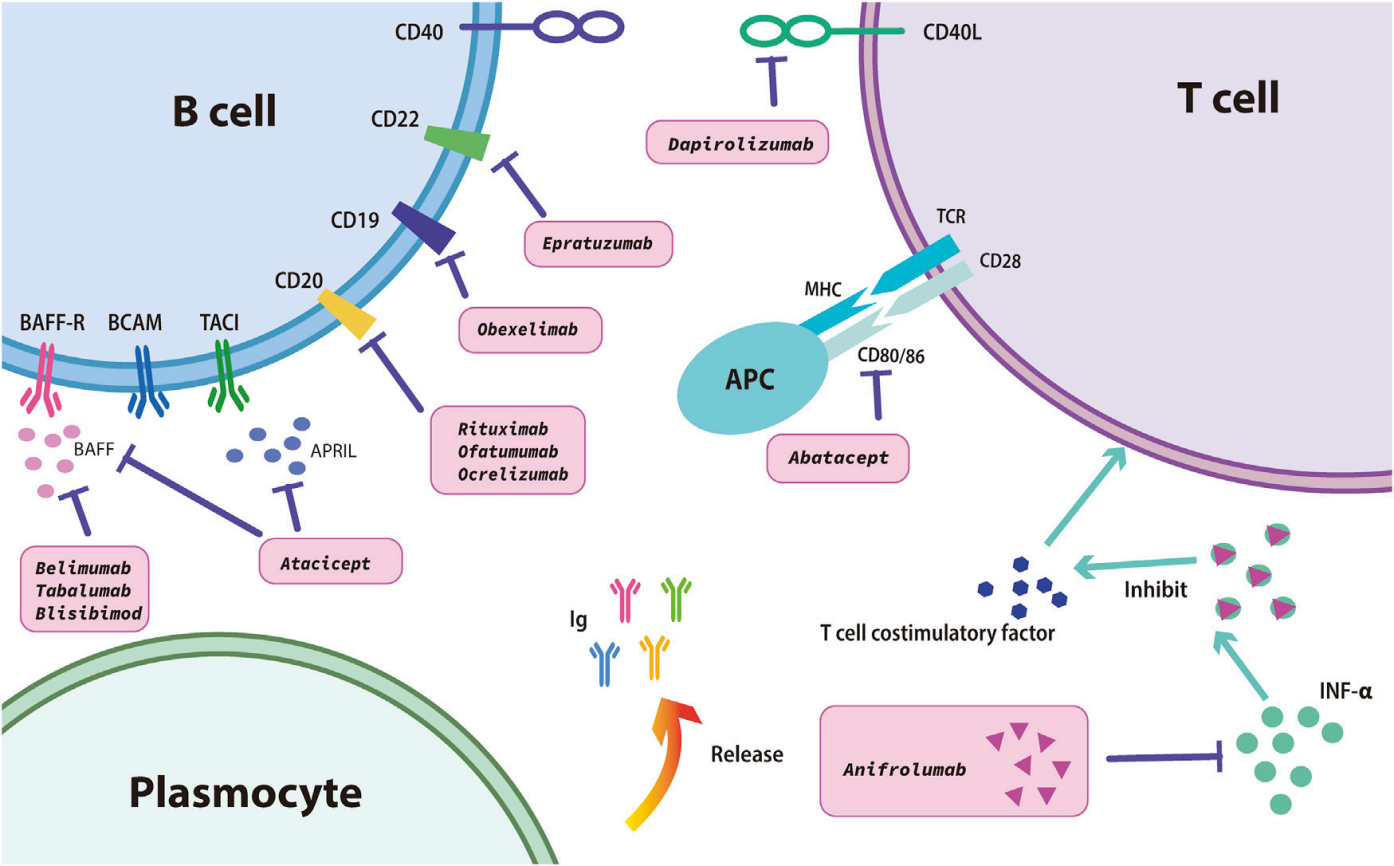
Mechanism of targeted drugs in the treatment of SLE.

B lymphocyte stimulator (BLyS), often referred to as B-cell-activation factor (BAFF), is a member of the tumor necrosis factor (TNF) cytokine superfamily and is essential for B-cell survival and development (Figure 1) (Blair and Duggan, 2018). SLE is associated with B-cell hyperactivity, autoantibodies, and increased concentrations of BLyS (Zhang et al., 2001).

Belimumab, a fully humanized anti-BAFF monoclonal antibody, has been the first biologic drug to receive a license for the treatment of SLE to date. This medication binds to BAFF and prevents soluble BAFF from attaching to B cell receptors, thereby preventing B cell survival, differentiation, and activation (Blair and Duggan, 2018). Belimumab was formally approved for the treatment of SLE through a phase III randomized controlled trial (RCT) in 2011. And its efficacy is demonstrated by four large double-blinded phase Ⅲ RCT (Furie et al., 2011; Navarra et al., 2011; Stohl et al., 2017; Zhang et al., 2018). In the RCT, this medicine was shown to be effective in reducing SLE disease activity and severe flares and delaying the onset of lupus in addition to standard therapy (Furie et al., 2011; Navarra et al., 2011). And according to the findings of a prospective cohort trial, belimumab may have clinical benefits for people with acute and subacute cutaneous lupus erythematosus by decreasing the frequency of flares and slowing the progression of skin lesions in patients with active SLE (Iaccarino et al., 2017). In a 104-week trial conducted at 107 sites in 21 countries, the probability of kidney-related events, morbidity, and mortality was all lower in the trial group compared to the control group, which demonstrates the utility of belimumab with standard therapy (Furie et al., 2020). The effectiveness, safety, and pharmacokinetics of intravenous belimumab were assessed in a Phase-2, randomized, placebo-controlled, double-blind research for children with systemic lupus erythematosus (cSLE). The results show that belimumab was well tolerated by paediatric patients, and the pharmacokinetics, pharmacodynamics and safety profiles were similar to those of adults with SLE (Brunner et al., 2020). A 28-week open-label study found that increases occurred in the proportions of patients achieving primary efficacy renal response and complete renal response from open-label baseline to week 28. Additionally, no new safety signals were identified, and efficacy was generally maintained throughout the open-label phase (Furie et al., 2022).

#### 2.1.2 Tabalumab

Similar to belimumab, tabalumab is a fully human IgG4 monoclonal antibody against soluble and membrane BAFF. Two phase Ⅲ randomized, multicenter, double-blinded, placebo-controlled trials (ILLUMINATE-1 and ILLUMINATE-2) were taken to assess efficacy and safety of subcutaneous tabalumab in patients with SLE. The results of the study demonstrated that the key clinical efficacy endpoints of ILLUMINATE-1 failed to achieve statistical significance, and the safety profile of tabalumab was similar to placebo (Isenberg et al., 2016). However, ILLUMINATE-2 met the primary endpoint of SLE response index 5 in the cohort receiving tabalumab 120 mg every 2 weeks (Merrill et al., 2016).

Although both studies noted that there were no serious safety signals, LUMMINATE-2 revealed that more patients (8.5%) receiving tabalumab than placebo reported depression and 3 patients in the tabalumab cohort attempted suicide while on treatment (Merrill et al., 2016). Although the results of the two phase Ⅲ trials of tabalumab were unsatisfactory, they did not indicate little or no efficacy. That may be a result of tabalumab’s researchers skipping a phase Ⅱ clinical trial and going directly to a phase Ⅲ trial.

#### 2.1.3 Blisibimod

Blisibimod is a potent and selective inhibitor of BAFF and has a unique tetravalent, ‘peptibody’ structure features. The results of randomized, double-blind phase 1a and phase 1b trials demonstrated that the safety and tolerability profile of blisibimod in SLE was comparable with that of placebo (Stohl et al., 2015). Subsequently, a phase 2 RCT in patients with moderate-to-severe SLE was taken. In this RCT, SLE responder index 5 response rates were not significantly improved in the pooled blisibimod groups compared with placebo; however, reductions in proteinuria, changes in anti-double standard DNA antibody, complement C3 and C4, and reductions in B cells were observed significantly with blisibimod (Furie et al., 2015). Therefore, the phase Ⅲ RCT of blisibimod enrolled patients with high disease activity. Although the SLE responder index 6 was not met in the study, blisibimod was well-tolerated and was associated with steroid reduction, decreased proteinuria and biomarker responses (Merrill et al., 2018a).

#### 2.1.4 Atacicept

Atacicept, a fusion protein of the transmembrane activator calcium moderator and cyclophilin lingand interactor (TACI) receptor and IgG, inhibit both BAFF and a proliferation-inducing ligand (APRIL). APRIL is another cytokine that has been identified as an important B-cell regulator. BAFF and APRIL levels are increased in patients with SLE, which indicates that dual blockade by atacicept may be more effective than blockading BAFF alone (Bag-Ozbek and Hui-Yuen, 2021).

A phase Ⅱ b RCT of 24-week, multicenter, randomized, double-blind, placebo-controlled, parallelism was taken to evaluate the safety and efficacy of atacicept in patients with SLE. The results of the study showed that there was a trend toward an improved SLE responder index 4 response rate with atacicept 75 mg and 150 mg as compared with placebo. Additionally, the risk of serious adverse events and serious or severe infection was not increased with atacicept as compared with placebo (Merrill et al., 2018b). The *post hoc* analyses of phase Ⅱ b RCT demonstrated that low disease activity (LDA) and lupus low disease activity state (LLDAS) were attainable at week 24 in the patients with high disease activity (HDA) receiving atacicept 150 mg compared to placebo (Morand et al., 2020b). Additionally, patients on continuous 150 mg atacicept had a reduced risk of first severe flare and a longer time to first severe flare. The study has drawn the conclusion that long-term treatment with atacicept 150 mg in SLE patients had an acceptable safety profile and durable efficacy (Wallace et al., 2021).

#### 2.1.5 Rituximab

Rituximab (RTX) is a chimeric monoclonal antibody that targets the B-cell CD20 (Figure 1) (Durcan et al., 2019). CD20 is a cell-surface antigen expressed on most B cells, except plasma cells, the majority of plasmablasts, and lymphoid stem cells (Gelfand et al., 2017; Payandeh et al., 2019). RTX was initially developed and approved by the Food and Drug Administration (FDA) for the treatment of non-Hodgkin’s lymphoma and has successfully entered rheumatology with benefits in the treatment of rheumatoid arthritis (RA) and antineutrophil cytoplasmic antibody (ANCA)-associated vasculitis (Stone et al., 2010; van Vollenhoven et al., 2015). RTX was first used in SLE in 2002, and 5/6 patients with refractory lupus erythematosus had a clinical response to the combination of RTX, CYC, and high-dose corticosteroids (Leandro et al., 2002). Subsequently, RTX is successful in treating refractory SLE symptoms such as nephritis and neuropsychiatric illness (Gottenberg et al., 2005; Leandro et al., 2005; Roccatello et al., 2011).

For the treatment of SLE, the combination of RTX and belimumab has undergone substantial research and appears to be a valuable potion for several clinical situations. Although RTX has a greater capacity to deplete B cells compared to belimumab, RTX’s SLE RCT has not achieved its primary clinical endpoint (Wise and Stohl, 2020). A randomized controlled trial was taken to evaluate the effectiveness of belimumab after RTX in SLE patients. In this study, patients were treated with RTX and then 4–8 weeks later were randomized (1:1) to receive belimumab or placebo for 52 weeks. The result of this study showed that belimumab after rituximab significantly reduced serum IgG anti-dsDNA antibody levels and reduced the risk of severe flare in SLE patients who are refractory to conventional therapy at 52 weeks (Shipa et al., 2021). The efficacy of the combination of RTX and belimumab is theoretically plausible but requires more clinical studies and case reports to confirm it.

#### 2.1.6 Ofatumumab

The main side event of RTX is allergic response because it is a chimeric anti-CD20 antibody. Therefore, other anti-CD20 antibodies, like ofatumumab, are investigated in SLE. Ofatumumab is a fully humanized anti-CD20 monoclonal antibody and is a safe, well-tolerated and effective alternative for B cell depletion (Cinar et al., 2021). Ofatumumab has recently received permission for treatment in individuals with chronic lymphocytic leukemia (Sandhu and Mulligan, 2015) and multiple sclerosis (Hauser et al., 2020). In a single-center retrospective case series of 16 patients treated with ofatumumab, 14/16 patients were well-tolerated. In this study, B cell depletion was achieved in 12 patients and was associated with improvement in serological markers of disease activity including ANA, anti-dsDNA antibody, and complement levels. Additionally, half of the patients with LN achieved renal remission by 6 months (Masoud et al., 2018). In an endogamous Pakistani kindred with monogenic SLE, 2/3 of the siblings had severe infusion-related reactions to RTX. Therefore, ofatumumab has been used and resulted in marked clinical improvement in both patients (Lei et al., 2018). A study of single-center retrospective case series for juvenile SLE found that significant clinical improvement was observed in all cases after using ofatumumab, mirrored by improved laboratory markers of disease activity including anti-dsDNA antibody, complement levels, and proteinuria (Cinar et al., 2021).

#### 2.1.7 Ocrelizumab

Ocrelizumab is another fully humanized anti-CD20 antibody. The CD20 epitopes binding ocrelizumab and RTX are overlapping. The result of a phase Ⅲ study showed that overall renal response rates with ocrelizumab were numerically superior to those with placebo. However, ocrelizumab in SLE patients with class III/IV LN was terminated prematurely due to a high rate of serious infections in the cohort treated with ocrelizumab (Mysler et al., 2013). Anyhow, initial results suggested ocrelizumab may have some efficacy in treating LN. And ocrelizumab was shown to have great efficacy in treating multiple sclerosis in several phase Ⅲ trials (Lamb, 2022).

#### 2.1.8 Obexelimab

CD19 is a transmembrane protein on the surface of B lymphocytes and is an important co-receptor for B cell antigen receptor (BCR) signaling. It is closely related to the activation, signal transduction, and growth of B cells. CD19 co-linked with BCR synergistically enhances calcium release, mitogen-activated protein kinase activity and cell proliferation (Li et al., 2017). CD19 has been widely used in the diagnosis and prognosis of leukemia, lymphoma, and immune system diseases, and is an important target for immunotherapy. Fcγ receptor (FcγR) IIB is a low-affinity IgG receptor expressed on the surface of B cells, which, when combined with the Fc segment of IgG, crosslinks with the BCR, raises the threshold of B cell activation, reduces antibody production, and thus plays a negative feedback regulatory function in humoral immunity (Espéli et al., 2016).

XmAb5871 (also known as obexelimab), a monoclonal antibody targeting CD19 and FcγRIIb, was found to inhibit calcium transport, proliferation, and co-stimulatory molecule expression in B cells in SLE patients and healthy volunteers, thereby suppressing humoral immunity and reducing IgM, IgG and IgE levels (Horton et al., 2011). The result of a phase Ⅱ study showed that XmAb5871 was well-tolerated, the infection rate was low, and flares were similar to other trials, which supports further evaluation of XmAb5871 in SLE (Merrill et al., 2018c).

#### 2.1.9 Epratuzumab

CD22 is expressed in mature B cells and modulates activation and migration of B cells by acting as an inhibitory co-receptor of the B-cell receptor. Epratuzumab is a humanized antibody binding to the glycoprotein CD22 of the cell surface of mature B cells (Clowse et al., 2017).

Initially, two phase Ⅲ study (ALLEVIATE-1 and -2) was taken to evaluate the efficacy and safety of epratuzumab in patients with moderate/severe flaring SLE. However, both initial ALLEVIATE studies were discontinued prematurely because of the drug supply (Wallace et al., 2013). Subsequently, phase Ⅲ study (EMBLEM) with 227 patients showed that proportion of responders was higher in all epratuzumab groups than with placebo (Wallace et al., 2014). An open-label extension study indicated that open-label epratuzumab treatment was well tolerated for up to 3.2 years, and associated with sustained improvements in disease activity along with a reduction of corticosteroid dose (Wallace et al., 2016). This result has been confirmed in several other studies (Strand et al., 2014; Tsuru et al., 2016). On the contrary, results from two phase Ⅲ trials showed that patients treated with epratuzumab + standard therapy did not result improvements in response rates over that observed in the placebo + standard therapy group (Clowse et al., 2017).

### 2.2 Targeted drugs against T lymphocytes

Studies have shown that the production of anti-DNA antibodies and other pathogenic autoantibodies in SLE patients or mice is T-cell dependent (Comte et al., 2015; Shan et al., 2020). SLE has multiple T-cell abnormalities, and the activation of auto-reactive T lymphocytes and the resulting autoimmune responses are central to the occurrence of the disease (Yasutomo, 2003).

#### 2.2.1 Abatacept

Abatacept is a fusion protein composed of cytotoxic T-associated protein 4 (CTLA-4) (a leukocyte differentiation antigen) and the Fc region of IgG1 (an immunoglobulin fused to extracellular structures). This medicine could regulate T cell costimulatory molecules by interrupting the interaction between CD80/CD86 and CD28 (Figure 1) (Pimentel-Quiroz et al., 2016). Two signals are required for T cells to activate and attack antigens to produce an immune response. For the first signal, antigen-presenting cells (APCs) bind to major histocompatibility complex (MHC) molecules and be presentd to T cell receptors on the T cell surface; for the second signal, APCs present B7 protein on their cell surface to CD28 protein on the T cell surface. Abatacept binds to B7-1 and B7-2 and blocks the signaling 2 pathway, preventing T cells from being activated.

However, a study showed that the phase II b clinical study of abatacept failed to meet the primary/secondary endpoints measured by the British Isles Lupus Assessment Group (BILAG) (Merrill et al., 2010a). In another clinical trial, a total of 298 patients were treated for 52 weeks to compare the efficacy and safety of intravenous (IV) abatacept. The result showed that although the primary endpoint was not met, abatacept showed evidence of biological activity (greater improvements in anti-double-stranded DNA antibody, C3, and C4 levels and a 20%–30% greater reduction in mean urinary protein-to-creatinine ratio compared with placebo) and was well tolerated in patients with active class III or IV lupus nephritis (Furie et al., 2014).

These unsatisfactory results may be related to a flawed study design that requires continuous optimization at a later stage. The safety profile of abatacept in patients with SLE suggests that abatacept may be considered as an alternative in refractory cases.

#### 2.2.2 anti-CD40 antibody

Interaction between the CD40 ligand (CD40L, CD154; mainly expressed on activated T cells and platelets) and the CD40 receptor (expressed on a variety of cells, including antigen-presenting cells and B cells) is essential for the activation of the adaptive immune system and drive the pathological process in SLE, including B cell differentiation and proliferation (Furie et al., 2021).

In human SLE, CD40L has long been a desirable therapeutic target. Dapirolizumab is a polyethylene glycol-conjugated Fab’ fragment that targets CD40 ligand (Narain et al., 2020). Since then, two phase Ⅰ clinical studies have investigated dapirolizumab. The first study showed predictable pharmacokinetics in both healthy individuals and patients with SLE and was well tolerated, with no safety signals of concern (Tocoian et al., 2015). The second study showed that multiple doses of dapirolizumab were well-tolerated, and there were no thromboembolic events during the study (Chamberlain et al., 2017). A phase Ⅱ study was taken to investigate the dose response, efficacy, and safety of dapirolizumab. The result of the study showed that although the primary objective was not met, dapirolizumab appeared to be well tolerated, and patients exhibited improvements across multiple clinical and immunological measures of disease activity after 24 weeks relative to placebo (Furie et al., 2021). Further research is necessary to determine the clinical benefit of dapirolizumab.

#### 2.2.3 Inhibition of syk and CaMKIV

Spleen tyrosine kinase (Syk) and calcium/calmodulin kinase IV (CaMKIV), small molecule inhibitors of kinases, are aberrantly expressed in immune cells of SLE patients and may present novel treatment prospects.

Syk is implicated in membrane-mediated signal transduction in a variety of cells and overexpressed in T cells of patients with SLE (Krishnan et al., 2008; Grammatikos et al., 2013). In the MRL/lpr mouse model, the Syk inhibitor, R788, avoided the onset of skin disease and dramatically reduced established skin disease, with clinical effects lasting at least 1 month after discontinuation of the drug. Syk inhibitor also decreased the growth of spleen and lymph nodes and inhibited kidney disease progression (Deng et al., 2010).

When the combination of T cell receptor and CD3 is activated, CaMKIV, which is expressed at high levels in SLE T cells, translocates to the nucleus and results in aberrant T cell function. A study using the CaMKIV inhibitor KN-93 to treat MRL/lpr mice found that CaMKIV inhibitor in MRL/lpr mice significantly suppressed nephritis and dermatosis, reduced the expression of co-stimulatory molecules CD86 and CD80 on B cells, and prevented the generation of inhibition of interferon (IFN)-γ and TNF-α. In human SLE T cells, the silence of CaMKIV results in the inhibition of IFN-γ production, which confirms the rationality of developing small molecule CaMKIV inhibitors for the treatment of SLE patients (Ichinose et al., 2011).

### 2.3 Targeted drugs against cytokines

Patients with SLE have significantly elevated genetic markers of type I IFN, a cytokine involved in the inflammatory response. Type 1 IFN has long been implicated in the pathogenesis of lupus (Rönnblom et al., 2011). IFN-α is associated with SLE and its disease activity (Nikpour et al., 2008; Bauer et al., 2009), which can promote the development of various immune cells, lead to the expression of BAFF, upregulation of T cells, and inactivation of T regulatory cells.

#### 2.3.1 Anifrolumab

Anifrolumab, a monoclonal antibody that targets the type I IFN receptor, successfully treated individuals with SLE in a phase 3 randomized trial in 2020 (Morand et al., 2020a). The results showed that adverse events in patients in the anifrolumab group included respiratory infections, nasopharyngitis, and infusion-related reactions. The number of adverse outcomes was lower than that in the placebo group, as well as better GC tapering and severity of skin disease than with the placebo (Morand et al., 2020a). A phase Ⅱ study was taken to assess the efficacy and safety of anifrolumab. Although the result showed that the primary endpoint was not met, anifrolumab intensified regimen was associated with numerical improvements over the placebo acrossing endpoint, including complete renal response (CRR), urine protein-creatinine ratio, CRR with inactive urinary sediment, and sustained glucocorticoid reductions (Jayne et al., 2022). Still, more studies are required to confirm the effectiveness and safety of this drug.

#### 2.3.2 Sifalimumab

Sifalimumab is a human-derived anti-IFN-α antibody. A recent phase II b clinical trial demonstrated that Sifalimumab has an inhibitory effect on IFN gene expression in patients with moderately to severely active SLE, resulting in remission of disease activity and joint injury (Takeuchi et al., 2020). Therefore, it is important to further explore the role of the type I IFN signaling pathway in autoimmune diseases to develop more effective and safe therapies approaches.

Cytokines play a role in the pathogenesis of SLE, for example, IL-4, IL-5, IL-6, IL-10, and IL-15 are increased in the serum of SLE patients. The pathogenesis of SLE is still unclear, and multi-level analysis of relevant cytokines is beneficial to provide a basis for new target therapy for SLE in the future.

## 3 Natural products

**FIGURE 2:**
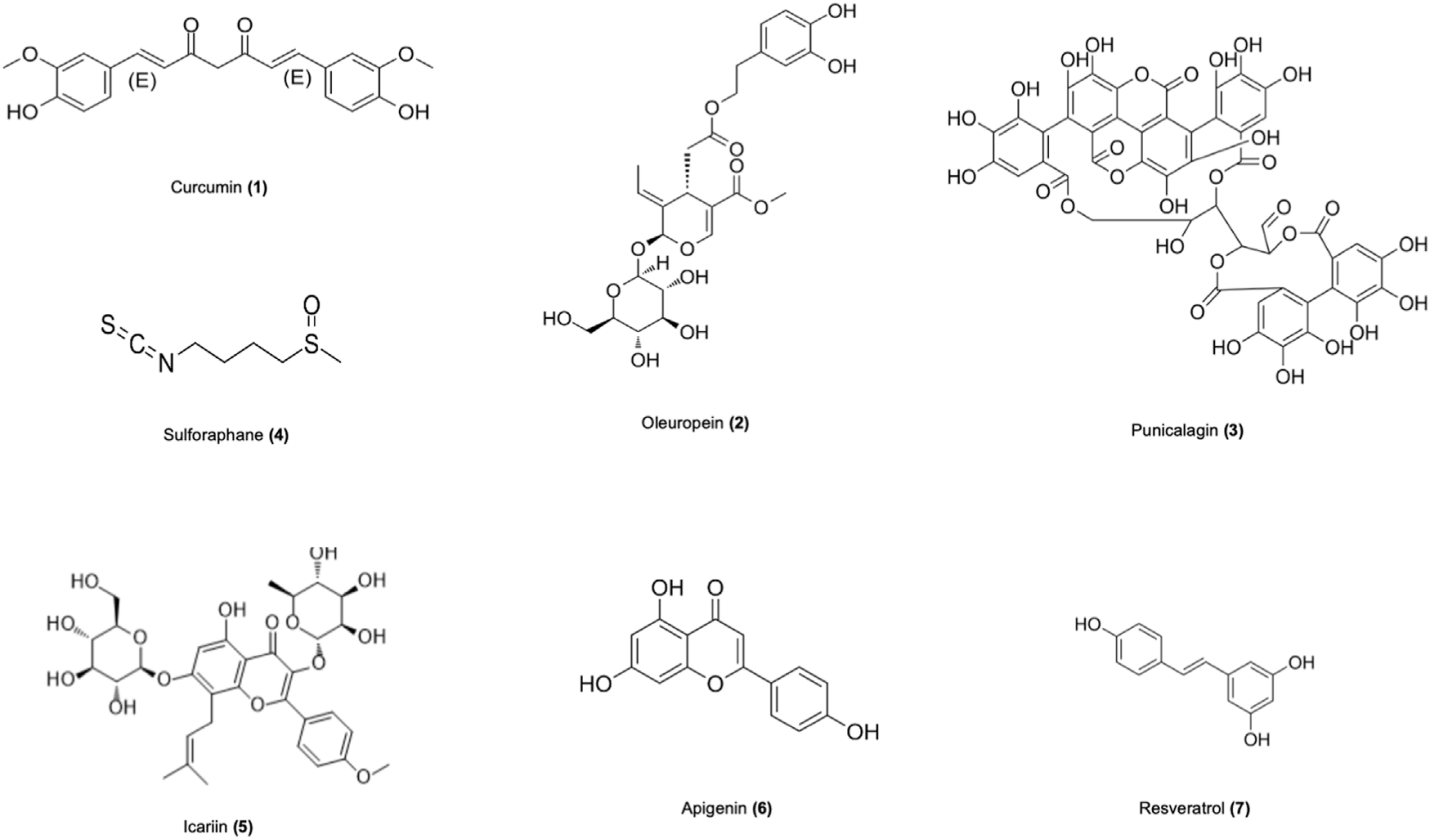
Chemical formulas for natural products effective in the treatment of SLE.

Numerous studies in recent years have revealed that natural products derived from animals and plants are effective in healing human diseases. Nature products showed promising further therapeutic effects in vitro and *in vivo* models of SLE through various mechanisms (Figure 2), as shown in Table 2. The mechanisms of nature products in the treatment of SLE are diverse and complex, which urgently need to be summarized and analyzed.

**TABLE 2:** The major effects of natural products in *vitro* and *in vivo* models of SLE.

Nature products	Animal/Cell model/SLE population	Dose	Duration	Therapeutic effects	Ref
CUR	MRL/lpr mice	200 mg/kg	8 weeks	Reduced proteinuria, renal inflammation, serum anti-ds DNA and spleen size	Zhao et al. (2019)
	PBMCs	0.1 μg/ml	48 h	Inhibite the expression and activation of PYK2 in PBMCs from LN patients rather than healthy subjects	Wang et al. (2017)
	NZB/W F1 mice	500 mg/kg	14 days	Reduced spleen weight and renal injury at 26 weeks of age. Reduced renal injury at 32 weeks of age	Dent et al. (2020)
	SLE patients with relapsing or refractory course classified according to ISN/RPS	3 capsules (contained 500 mg turmeric, of which 22.1 mg was the active ingredient curcumin) daily	3 months	Significant decrease in proteinuria, systolic blood pressure and hematuria in post-turmeric supplementation	Khajehdehi et al. (2012)
	SLE patients with SLEDAI >3 and 25(OH)D level <30 ng/ml	60 mg daily	3 months	No significant differences in SLEDAI reduction, decreased serum levels of IL-6, and increased levels of TGF-𝛽1 serum among groups after the treatment	Singgih Wahono et al. (2017)
OL	BALB/c mice	100 mg/kg	24 weeks	Reduced renal damage and decreased serum matrix metalloproteinase 3 and prostaglandine E2 kidneys levels	Castejon et al. (2019)
PCG	NZB/W F1 mice	1 and 3 mg/kg	7 weeks	Alleviate kidney injury and splenomegaly and reduce proteinuria and renal ICAM-1 and VCAM-1 expression	Seo et al. (2020)
SFN	MRL/lpr mice	12.5 mg/kg	28 weeks	Ameliorate renal function	Jiang et al. (2014)
	MRL/lpr mice	82.9 μmol/kg	27 days	Decrease the percentages of plasma cells, Tfh cells, neutrophils, and dendritic cells	Du et al. (2022)
ICA	MLR/lpr mice	10 mg/kg	8 weeks	Decreased serum anti-ds DNA antibody, immune complex and renal disease	Su et al. (2018)
API	SNF1 mice	3,6,20 mg/kg	8 weeks	Suppressed and promoted apoptosis of the APCs, T and B cells	Kang et al. (2009)
RES	PIL mice	50,75 mg/kg	7 months	Attenuated glomerulonephritis, proteinuria and immunoglobulin deposition	Wang et al. (2014)
	MRL/lpr mice	20 mg/kg	6 weeks	Reduced serum autoantibodies, relieved lupus nephritis and disease onset, and prolonged survival	Jhou et al. (2017)
	ApoE Fas mice	0.01% in water	10 weeks	Exerted fewer atherosclerotic plaques	Voloshyna et al. (2016)
	BALB/c mice	25 mg/kg and 50 mg/kg with its bio-enhancer piperine	4 months	Mitigated renal manifestations	Pannu and Bhatnagar (2020)
	MRL/Mp-Faslpr mice	0.01% in ethanol	10 weeks	Increased mRNA levels of SIRT1, decreased vascular endothelial growth factor and CX3CL1 mRNA in the hippocampus	Kasselman et al. (2022)

Abbreviations: SLE: systemic lupus erythematosus; CUR: curcumin; OL: oleuropein; PCG: punicalagin; SFN: sulforaphane; ICA: icariin; API: apigenin; RES: resveratrol; PBMCs: peripheral blood mononuclear cells; PYK2: Proline-rich tyrosine kinase 2; LN: lupus nephritis; ICAM: intercellular adhesion molecule 1; VCAM: vascular cell adhesion molecule 1; Tfh: T follicular helper; APC: antigen-presenting cells; ISN/RPS: The International Society of Nephrology/Renal Pathology Society; SLEDAI: systemic lupus erythematosus disease activity index; SIRT: sirtuin.

### 3.1 Curcumin

Ginger is thought to have anti-inflammatory and antioxidant effects, and the health-promoting properties of ginger have been attributed to its rich phenolic phytochemicals, such as curcumin (CUR) (Jolad et al., 2004; Grzanna et al., 2005; Jolad et al., 2005). CUR, a phenolic component extracted from turmeric, belongs to the ginger family and has been shown to have immunomodulatory and anti-inflammatory effects in various diseases (Chamani et al., 2022). According to animal reseatch, CUR has the potential to cure a wide range of inflammatory diseases (Aggarwal and Harikumar, 2009).

LN is one of the most severe SLE side effects and a significant contributor to morbidity and mortality in SLE patients (Anders et al., 2020). Although immunotherapy considerably improves the prognosis of patients with SLE and nephropathy, several patients with LN eventually get end-stage renal disease (Faurschou et al., 2010). In a mouse model prone to lupus erythematosus, CUR-treated MLR/lpr mice displayed a significant decrease in proteinuria and renal inflammatory response, lower serum anti-ds DNA levels, and smaller spleens, as well as downregulation of nucleotide-binding domain (NOD) like receptor protein 3 (NLRP3) inflammasome (Zhao et al., 2019). Proline-rich tyrosine kinase 2 (PYK2) is a non-receptor protein tyrosine kinase belonging to the superfamily of adhesion kinases. It plays a significant role in the development of autoimmune disorders and delivers critical signals during lymphocyte activation (Sasaki et al., 1995). According to *in vitro* research, CUR can inhibit the proliferation of peripheral blood mononuclear cells in LN patients by suppressing the expression and activation of PYK2. Additionally, CUR inhibition showed a negative correlation with complement levels in the serum and positive correlation with proteinuria levels measured after 24 h, without affecting normal subjects (Wang et al., 2017). A RCT clinical study used CUR 60 mg combined with cholecalciferol as the treatment regimen for the trial group. The result of the study found that decreased levels of serum IL-6 have a positive correlation with Safety of Estrogens in Lupus Erythematosus National Assessment–Systemic Lupus Erythematosus Disease Activity Index (SLEDAI) reduction, and there were no significant differences in SLEDAI reduction, decreased serum level of IL-6, and increased levels of TGF-β1 serum among group after treatment (Singgih Wahono et al., 2017). A study in an experimental model of SLE found that CUR-treated SLE mice had lower spleen weight and renal injury compared to vehicle-treated SLE mice when treatment started at 26 weeks of age. When CUR treatment started at 32 weeks of age, renal injury was reduced in SLE mice (Dent et al., 2020).

Although CUR has potential therapeutic value for SLE, its poor bioavailability limits its therapeutic effect due to poor absorption, rapid metabolism, and rapid systemic elimination (Anand et al., 2007). One study found that reconstituting CUR with a non-curcumin-like component of turmeric can greatly improve the bioavailability of turmeric (Antony et al., 2008). An RCT study was taken to evaluate the effect of turmeric and the result showed that proteinuria, systolic blood pressure and hematuria decreased significantly in SLE patients after received capsules that contained turmeric for 3 months (Khajehdehi et al., 2012).

### 3.2 Oleuropein

Oleuropein (OL) is key to the main anti-inflammatory action of olive leaf extract and has a pharmacologically important antioxidant, anti-inflammatory, and immunomodulatory properties (Ryu et al., 2015; Qabaha et al., 2018). Peracetylated oleuropein (Per-OL) are acyl derivatives of OL and have the potential to protect membrane components due to their lipophilic nature, which enables them to cross cytoplasmic cell membranes and be taken up by cells. This results in better absorption through intestinal epithelial cell monolayers than OL (Stamatopoulos et al., 2013).

OL and Per-OL can significantly reduce renal injury and serum matrix metalloproteinase-3 and prostaglandin E2 levels. Studies have shown that in mouse models of SLE, the expression of nuclear factor E2-related factor 2 (Nrf2) and heme oxygenase (HO-1) antioxidant protein was upregulated in mice fed with OL and Per-OL diets. However, there was a significant improvement in the stimulation of Janus kinase/signal transducer and activator of transcription (JAK/STAT)**,** mitogen-activated protein kinases (MAPK), nuclear transcription factor-kappa B (NF-κB) and inflammasome nucleotide-binding domain, leucine-rich repeats-containing family, pyrin domain-containing-3 inflammatory vesicle pathway (Castejon et al., 2019). STAT3 signaling pathway is essential for the development of the Th17, and STAT3/IL-17 expression was upregulated in SLE patients (Chen et al., 2019). In peritoneal macrophages of SLE mice, it was discovered that OL and Per-OL diets decreased T helper (Th)1, Th2, and Th17 cytokines, as well as the protein expressions of p-STAT3, COX-2, and inducible nitric oxide synthase (iNOS). At the same time, NF-κB activation was inhibited (Castejón et al., 2020).

### 3.3 Punicalagin

Punicalagin (PCG), a major polyphenol-rich in pomegranate, has antioxidant, anti-inflammatory, and immunosuppressive effects (Venusova et al., 2021).

Studies have shown that PCG can effectively inhibit protease-activated receptor-2 (PAR2), which is abundantly expressed in human tissues, particularly in renal cells, such as podocytes, thylakoid cells, tubular epithelial cells and infiltrating immune cells (Vesey et al., 2007; D'Andrea et al., 1998). In the NZB/W F1 mouse model and human podocyte cell lines, PCG significantly reduced PAR2-mediated activation of extracellular signal-regulated kinases 1 and 2, NF-κB signaling pathways, intercellular adhesion molecule 1 (ICAM-1), and vascular cell adhesion molecule 1 (VCAM-1) as well as expression of IL-8, IFN-γ and TNF-α. Meanwhile, injection via PCG dramatically reduced renal injury and splenomegaly, decreased proteinuria, and decreased the expression of renal ICAM-1 and VCAM-1 expression in NZB/W F1 mice (Seo et al., 2020).

### 3.4 Sulforaphane

Sulforaphane (SFN), found in cruciferous plants, is an activator of NRF2 and has several biological properties that may lower the risk of developing chronic diseases, such as anti-inflammatory, antioxidant, and anti-tumor activities (Raiola et al., 2017).

SFN is an NRF2 agonist with positive effects in ameliorating oxidative stress. Previous research suggests that Nrf2 deficiency increases the development of LN, revealing a preventative or protective role for SFN. It found that Nrf2^−/−^ mice spontaneously develop lupus-like autoimmune nephritis at 60 weeks (Yoh et al., 2001). Experimental data have demonstrated the significance of anti-oxidative stress and the NF-kB pathway in lupus in a mouse model of LN (Jiang et al., 2014). SFN can act as an NRF2 activator to effectively ameliorate oxidative stress and inflammatory disease and suppress LN by inhibiting oxidative stress and NF-κB signaling pathway (Jiang et al., 2014).

Another study on the disease found that SFN improved the autoimmune response in the kidney of lupus-prone MRL/lpr mice. Th1 and Th17 cells expressed more and lived longer after receiving SFN therapy. The proportion of plasma cells, T follicular helper (Tfh) cells, neutrophils, dendritic cells, and quantity of malondialdehyde all decreased. Further studies revealed that the antioxidant activity of SFN was dependent on the activation of the peroxiredoxin 1 (PRDX 1) gene, which was increased in SFN-treated MRL/lpr mice (Du et al., 2022). The above studies also suggest that antioxidant therapy may be a potential approach to treating SLE.

### 3.5 Icariin

Icariin (ICA) is recognized as a traditional herb in eastern Asia, which benefits osteoporosis, chronic nephritis, asthma, hepatitis, and cardiovascular disease. ICA is the most abundant flavonol glycoside in Epimedium. It has a range of therapeutic effects, including anti-inflammatory, antioxidant, anti-cancer, and anti-aging properties (Li et al., 2015).

NF-κB is a crucial regulator of the production of several proteins linked to inflammation (Li and Verma, 2002). The severity of LN is directly correlated with NF-B activation, which is crucial for LN pathogenesis (Li et al., 2015). It was discovered that ICA treatment dramatically decreased serum urea nitrogen and creatine levels in MRL/lpr mice, and improved renal pathologies such as glomerular hyperplasia, glomerulosclerosis, and periglomerular inflammation. Further experiments revealed that ICA inhibited renal NF-κb activation, NLRP3 inflammatory vesicle activation, and TNF-α expression in MRL/lpr mice (Su et al., 2018). As a diagnostic biomarker for active LN, C-C motif chemokine ligand (CCL)2, also known as monocyte chemoattractant protein 1 (MCP-1), is employed (Gupta et al., 2016). CCL2 levels were significantly elevated in MRL/lpr mice, while CCL2 overexpression was reduced by ICA therapy (Su et al., 2018). These results suggest a protective effect of ICA on SLE.

### 3.6 Apigenin

Flavonoid subclass Apigenin (API) is chemically known as 4′,5,7 trihydroxyflavone. Flavonoids have various biological benefits include free radical scavenging, antioxidant, anti-inflammatory, and anti-cancer. Based on *in vivo*, and clinical trial studies, API is a potential therapeutic agent. This compound has the ability to reduce PG synthesis, nitric oxide generation, and the activity of numerous enzymes that control cell growth (Kasiri et al., 2018).

20 mg/kg of apigenin was daily administered into SNF1 mice with lupus-like lesions to watch the progression of severe nephritis. The results showed that API inhibited the response of Th1, Th17 cells, and B cells to major lupus autoantigens by suppressing autoantigen presentation and the value-added function of APCs. Additionally, API induced apoptosis in lupus T cells, B cells, and APCs via downregulating COX-2 expression (Kang et al., 2009).

### 3.7 Resveratrol

Resveratrol (RES) is a molecule known chemically as 35,4-trihydroxystilbene. It is a natural plant antitoxin that can be synthesized from various plants, including grapes, wine, soy, nuts, and chocolate, and is produced in pathogenic and stressful environments (Oliveira et al., 2017). The molecule can be distributed into tissues by reversible interaction with serum albumin by its cis-trans isomeric (Tang et al., 2017).

Treatment of pristane-induced lupus mice with different doses of RES has been shown to reduce proteinuria, renal immunoglobulin deposition, glomerulonephritis, and serum IgG1 and IgG2a levels. Additionally, CD4^+^ T cell proliferation and CD4^+^ T cell death were both decreased by RES, as well as the expression of CD69 and CD71 in CD4^+^ T cells. It caused CD4^+^ T cells to undergo apoptosis. CD4 IFN γ+ Th1 cells and Th1/Th2 cell ratio were decreased. In in vitro, antibody production and B-cell proliferation were also inhibited. The treatment of pristane-induced lupus mice with RES may inhibit CD4^+^ T cells by triggering the silent mating type information regulator 2 homolog 1 (SIRT1) (Wang et al., 2014). Further studies revealed that RES increased the expression of FcγRIIB in B cells of MRL/lpr mice, which led to a considerable decrease of plasma cells in the spleen and bone marrow, thereby reducing serum autoantibody titers. This may be due to the upregulation of FcγRIIB by affecting the increase of SIRT1 protein and p65 NF-κB deacetylation (Jhou et al., 2017). In the ApoE^−/−^Fas^−/−^ double knockout mouse model, RES attenuated atherosclerosis in the mouse model, with the RES-treated group generating fewer atherosclerotic plaques than the untreated group. Also, the data showed that RES inhibited cholesterol efflux in macrophages (Voloshyna et al., 2016). A study was taken to evaluate the combinatorial effect of RES and piperine on the murine model. Renal manifestations (proteinuria and decreased creatinine in urine) were successfully mitigated by the combination of RES and piperine (Pannu and Bhatnagar, 2020). In a RES-treated atherosclerosis-prone lupus mouse model, RES tends to increase mRNA levels of sirtuin 1, and decrease vascular endothelial growth factor and CX3CL1 (neurotactin ligand) mRNA in the hippocampus. The study demonstrated that RES could potentially be a therapeutic candidate in the modulation of cognitive dysfunction in neuropsychiatric lupus, especially motor incoordination. (Kasselman et al., 2022).

RES also has some limitations. Its metabolism in in vivo is rapid and its bioavailability is limited (Walle, 2011; Pujara et al., 2017). It also shows some mild toxic effects at high doses of treatment, such as headache, lower extremity myalgia, drowsiness, blood electrolyte changes, and rash (Cottart et al., 2010). Therefore, its bioavailability and toxicity should be taken into account when considering its possible therapeutic effects.

## 4 Conclusion

In this review, we enumerate the great potential of antibody drugs and natural medicines in the treatment of SLE. Despite the great therapeutic value of targeted and natural drugs, there are still some limitations in the treatment of autoimmune diseases. Therefore, every effort should be made to develop safer and more effective therapies, develop treatment regimens that rapidly control lupus activity, prevent future relapses, and prevent additional short-term and long-term complications.
